# Statins Decrease Programmed Death-Ligand 1 (PD-L1) by Inhibiting AKT and β-Catenin Signaling

**DOI:** 10.3390/cells10092488

**Published:** 2021-09-20

**Authors:** Woo-Jin Lim, Mingyu Lee, Yerin Oh, Xue-Quan Fang, Sujin Lee, Chang-Hoon Lim, Jooho Park, Ji-Hong Lim

**Affiliations:** 1 Department of Biomedical Chemistry, College of Biomedical & Health Science, Konkuk University, Chungju 27478, Korea; lwj0908@kku.ac.kr (W.-J.L.); lydia9063@kku.ac.kr (Y.O.); gkrrnjs654852@kku.ac.kr (X.-Q.F.); 210341532@kku.ac.kr (S.L.); lchoo1196@kku.ac.kr (C.-H.L.); pkjhdn@kku.ac.kr (J.P.); 2 Department of Applied Life Science, Graduate School, BK21 Program, Konkuk University, Chungju 27478, Korea; 3 Division of Allergy and Clinical Immunology, Brigham and Women’s Hospital and Department of Medicine, Harvard Medical School, Boston, MA 02115, USA; leemk08@gmail.com; 4 Diabetes and Bio-Research Center, Konkuk University, Chungju 27478, Korea

**Keywords:** statins, PD-L1, immune checkpoint inhibitor, AKT, β-catenin

## Abstract

Retrospective observational studies have reported that statins improve clinical outcomes in patients previously treated with programmed cell death protein 1 (PD-1)-targeting monoclonal antibodies for malignant pleural mesothelioma (MPM) and advanced non-small cell lung cancer (NSCLC). In multiple mouse cancer models, de novo synthesis of mevalonate and cholesterol inhibitors was found to synergize with anti-PD-1 antibody therapy. In the present study, we investigated whether statins affect programmed death-ligand 1 (PD-L1) expression in cancer cells. Four statins, namely simvastatin, atorvastatin, lovastatin, and fluvastatin, decreased PD-L1 expression in melanoma and lung cancer cells. In addition, we found that AKT and β-catenin signaling involved PD-L1 suppression by statins. Our cellular and molecular studies provide inspiring evidence for extending the clinical evaluation of statins for use in combination with immune checkpoint inhibitor-based cancer therapy.

## 1. Introduction

An increasing number of studies have demonstrated that abnormal de novo synthesis of mevalonate and cholesterol are closely associated with tumor development, growth, and progression [1]. Cholesterol, an essential component of the cell membrane and lipid rafts, is closely associated with oncogenic growth signaling [2]. Cholesterol can activate oncogenic hedgehog signaling by interacting with the smoothened receptor, which belongs to the G protein coupled receptor (GPCR) [3,4]. In addition, oncogenic growth-signaling cascades, including phosphoinositide 3-kinase (PI3K)-protein kinase B (AKT), mitogen-activated protein kinase (MAPK), CD44, c-Src, and c-Met, which are enriched in lipid rafts and related to both cancer development and progression, are tightly controlled by intracellular cholesterol levels [2,5]. Statins are cholesterol-lowering drugs that selectively inhibit HMG-CoA reductase (HMGCR), the rate-limiting enzyme of the mevalonate biosynthesis pathway, and are widely used for the treatment of hyperlipidemia and cardiovascular diseases [6]. Many preclinical and clinical studies have shown that abnormally increased cholesterol levels are associated with a higher cancer incidence and mortality, and cholesterol-lowering drugs, such as statins, exhibit beneficial effects in reducing the risk of cancer incidence and cancer-related mortality [2]. Several observational studies have demonstrated that statins have beneficial effects, including improving overall survival and reducing the risk of cancer-related mortality in patients with multiple types of cancers [7] such as prostate [8], colorectal [9], renal cell carcinoma [10], breast [11] and ovarian cancer [12], and lymphoma [13]. In addition, synergistic anti-cancer effects of statins on chemotherapy and targeted therapy in patients with various cancer types have been observed. Phase I/II clinical evaluations of statins, in combination with various standard-of-care therapies such as gefitinib, afatinib, sorafenib, and gemcitabine in non-small cell lung cancer (NSCLC), hepatocellular carcinoma, and pancreatic cancer, have been performed [14,15,16,17]. Notably, recent evidence has indicated that statins lead to better clinical outcomes in malignant pleural mesothelioma (MPM) and advanced NSCLC patients treated with the immune checkpoint inhibitor, nivolumab, a programmed cell death protein 1 (PD-1)-targeting monoclonal antibody, indicating that statins are also available for combination cancer therapy with immune checkpoint inhibitors [18,19]. However, extended preclinical and clinical evaluations on how statins can improve the therapeutic efficacy of immune checkpoint inhibitors for cancer treatment are necessary for clinical application. Mechanistically, statins suppress the enzymatic activation of Ras and Rho proteins, which play a central role in cancer development by inhibiting the prenylation of Ras and Rho proteins [20]. Farnesyltransferases (FTase) and geranylgeranyltransferases (GGTase) involve the transfer of isoprenoid chains such as geranylgeranyl pyrophosphate (GGPP) and farnesyl pyrophosphate (FPP), which are synthesized during de novo synthesis of mevalonate and cholesterol, to the carboxyl-terminal cysteine of the target proteins, and this chemical reaction is called protein prenylation [21]. Accumulated evidence has shown that FTase and GGTase inhibitors (TFIs and GGTIs, respectively) attenuate tumor growth and progression by suppressing oncogenic growth signal transduction, cell cycle progression, proliferation, and cell survival [22].

T-cell-mediated immunity primarily regulates the elimination of pathogens and abnormally transformed tumor cells to maintain homeostasis in the body [23]. Programmed death-ligand 1 (PD-L1) is encoded by the *CD274* gene and a major co-inhibitory checkpoint molecule [24]. PD-L1 physically interacts with programmed cell death protein 1 (PD-1), which is predominantly expressed in T-cells and suppresses the T-cell-mediated elimination of tumor cells [24]. Multiple types of tumor cells, including those associated with lung cancer, breast cancer, and melanoma, highly express PD-L1 and signals to T-cells called “Don’t kill me” via PD-L1/PD-1 interaction [23]. Considering that tumor cells can evade T-cell immunity through interaction with PD-L1/PD-1, various immune checkpoint inhibitors with monoclonal antibodies blocking the interaction of PD-L1/PD-1 that consequentially inhibit tumor cell evasion from T-cells have been developed [25]. For instance, PD-L1-neutralizing monoclonal antibodies, namely atezolizumab, avelumab, and durvalumab, were developed and are widely used in single or combination treatment for patients with metastatic NSCLC, triple-negative breast cancer (TNBC), advanced renal cell carcinoma, metastatic melanoma, ovarian cancer, esophageal cancer, mantle cell lymphoma, diffuse large B-cell lymphoma, and follicular lymphoma [26]. The expression of the PD-L1 gene and protein is regulated by various biological processes, such as genomic alteration, epigenetic modification, transcription, and post-transcriptional and post-translational modifications [23]. Upregulated *CD274* through genomic amplification and translocation was identified in primary mediastinal large B-cell lymphoma (PMBCL), NSCLC, and gastric adenocarcinoma, consequently leading to immune escape [27,28,29]. Furthermore, recent reports have shown that bromodomain and extraterminal (BET) proteins as well as histone deacetylase (HDAC) transcriptionally activate and inactivate *CD274* expression, respectively, via epigenetic regulation [30,31]. An increasing amount of evidence has supported the fact that various oncogenic transcription factors, such as MYC, the signal transducer and activator of transcription 3 (STAT3), hypoxia-inducible factor1α (HIF1α), nuclear factor-κB (NF-κB), and β-catenin, directly bind to the *CD274* promoter and activate *CD274* gene expression [23]. Hyperactivation by genomic mutations in oncogenic growth-signaling molecules, such as MAPK, PI3K, and the epidermal growth factor receptor (EGFR), can also enhance *CD274* expression in malignant melanoma, NSCLC, and gallbladder cancer [32,33,34]. Indeed, a selective small molecule inhibitor of BRAF has been found to sensitize patients with BRAF-mutated melanoma to achieve therapeutic efficacy [35]. Therefore, these observations indicate that small molecules which suppress PD-L1 expression in cancer cells are beneficial for PD-1 and PD-L1-neutralizing antibody-based cancer treatment.

## 2. Materials and Methods

### 2.1. Cell Culture and Reagents

Lung (NCI-H23, A549, NCI-H358, Calu-1, NCI-H460, and NCI-H1299), breast (SK-BR-3, MCF-7, and MDA-MB-231), colorectal (HCT116), hepatocellular (SK-HEP-1), and cervical (HeLa) cancer cells were obtained from the Korean Cell Line Bank (Seoul, Korea). Melanoma cell lines (A375 and A2058) were obtained from the American Type Culture Collection (Manassas, VA, USA). Cells were cultured in 10% fetal bovine serum (FBS) as well as penicillin/streptomycin-contained Dulbecco’s modified Eagle’s medium (DMEM) and Roswell Park Memorial Institute (RPMI) 1640 at both 20% O2 and 5% CO2. Cell culture medium, FBS, and antibiotics were purchased from HyClone Thermo Scientific (Waltham, MA, USA). Simvastatin (S6196), Lovastatin (438185), Lonafarnib (SML1457), and GGTI-2133 (G5294) were purchased from Sigma Aldrich (St. Louis, MO, USA). Atorvastatin (S5715), Fluvastatin (S2061), and MK-2206 (S1078) were obtained from Selleckchem (Houston, TX, USA). All chemicals stock solution was dissolved in dimethyl sulfoxide (DMSO).

### 2.2. Protein Extraction and Western Blotting

To analyze protein expression, cells were washed by cold phosphate-buffered saline (PBS) and lysed by using 1% NP-40 lysis buffer containing 150 mM NaCl, 5 mM ethylenediaminetetraacetic acid (EDTA), 50 mM Tris-HCl (pH7.4), protease inhibitor, and phosphatase inhibitor cocktail. Protein contents were normalized using the Bradford protein assay. Total proteins (30 μg) were separated following the molecular weight through sodium dodecyl sulfate-polyacrylamide gel electrophoresis (SDS-PAGE) and then proteins were transferred onto polyvinylidene difluoride (PVDF) membrane (Millipore, Burlington, MA, USA). Transferred proteins on the PVDF membrane were reacted with primary antibodies for 12 h at 4 °C and secondary antibodies for 1 h at room temperature. Proteins incubated with primary and secondary antibodies were washed by using TBST (tris-buffered saline, 0.1% TWEEN 20) to remove non-specific reactions between target proteins and antibodies. Target proteins’ expression, such as for PD-L1, β-actin, phospho-ERK1/2, ERK1/2, phospho-AKT-S473, AKT, phospho-β-catenin-S552, and β-catenin, were analyzed by using the Enhanced Chemiluminescence (ECL) Prime kit (GE Healthcare, Pittsburgh, PA, USA). For western blotting, antibodies against anti-PD-L1 (CST-29122), anti-β-actin (sc-47778), anti-phospho-ERK1/2 (CST-4370), anti-ERK1/2 (sc-94), anti-phospho-AKT-S473 (CST-4060), anti-AKT (CST-4691), anti-phospho-β-catenin-S552 (CST-9566), and anti-β-catenin (CST-8480) were purchased from Cell Signaling Technology (Danvers, MA, USA) and Santa Cruz Biotechnology (Dallas, TX, USA).

### 2.3. RNA Isolation, cDNA Synthesis, and RT-PCR

Total RNA was isolated with TRIzol (Invitrogen, Carlsbad, CA, USA) and 2 µg of total RNA was used for cDNA synthesis using a high capacity cDNA reverse transcription kit (Applied Biosystems, Foster City, CA, USA). Purity of isolated total RNA was measured by using the ratio of absorbance at 260 nm and 280 nm (A260/A280), and the ratio of A260/A280 higher than 1.8 was considered to be of acceptable purity. To analyze the gene expression, quantitative PCR was performed using the SYBR Green PCR Master Mix (Applied Biosystems, Foster City, CA, USA) and LightCycler96 real-time PCR (Roche, Basel, Switzerland). Ct values were normalized to the human *36B4* gene and relative mRNA expression was calculated versus human *36B4* expression as previously described [36]. The primer sequences used in the experiment are shown in Table 1.

### 2.4. Chromatin Immunoprecipitation (ChIP)

A chromatin immunoprecipitation (ChIP) assay was performed by using the EZ-ChIP assay kit (Millipore, Burlington, MA, USA). A2058 and Calu-1 cells were incubated with 10 μM of simvastatin or DMSO in the absence or presence of 25-hydroxycholesterol (25-HC) for 16 h, and then cells were fixed in 1% formaldehyde-contained DMEM for 15 min. The fixed cells were lysed in ChIP-lysis buffer containing sodium dodecyl sulfate (SDS), protease inhibitor cocktail, and PMSF, and then cell lysates were sonicated by using an ultrasonic homogenizer (Bandelin Electronic, Berlin, Germany) for four cycles of 5 min (30 s on, 30 s off upon 30% of power). Samples were incubated with normal rabbit IgG or anti-β-catenin antibody for 16 h at 4 °C and then the target chromatin complex was obtained by using protein A and G agarose bead (Millipore, Burlington, MA, USA) pre-blocked with salmon sperm DNA (Millipore, Burlington, MA, USA). Samples were washed with buffer containing 150 mM NaCl (low salt wash buffer), 500 mM NaCl (high salt wash buffer), and 250 mM LiCl. Immunoprecipitated DNA was isolated by using a phenol:chloroform:isoamyl alcohol (25:24:1) as previously described [37] and then DNA was both amplified and analyzed by using the LightCycler96 real-time PCR (Roche, Basel, Switzerland). The PCR primer sequences for the *CD274* promoter were 5′-ATGTAGCTCGGGATGGGAAGT-3′ (forward) and 5′-TGTGTGTGTGTGTATGGGTGTA-3′ (reverse) as described previously [38].

### 2.5. Bioinformatic Analysis

β-catenin target genes’ expression, such as for *CCND1*, *BIRC5*, *ENC1*, *MMP7*, *AXIN2*, *BCL2*, and *TP53*, in pravastatin, simvastatin, lovastatin, or fluvastatin-treated lung (Calu-1) and pancreatic (PANC1 and MiaPaCa2) cancer cells were obtained from publicly available microarray datasets (GSE47458 and GSE149566). Microarray datasets were downloaded from the Gene Expression Omnibus (www.ncbi.nlm.nih.gov/geo, accessed on 1 August 2021) and analyzed to compare mRNA levels of β-catenin target genes between vehicle control and statins-treated lung and pancreatic cancer cells. Data are represented as means ± standard error (SE). Statistical analyses were conducted using Student’s *t*-test and one-way ANOVA post hoc test. Statins-treated samples were compared to the vehicle control.

### 2.6. Statistical Analysis

Illustrative figures were generated by using GraphPad Prism version 5 (GraphPad Software Inc., San Diego, CA, USA). Gene expression and chromatin immunoprecipitation (ChIP) experiments were performed in triplicates. Data are represented as means ± standard error (SE) and means ± standard deviations (SD). Statistical analyses were conducted using Student’s *t*-test for two experimental comparisons and one-way ANOVA post hoc test for multiple comparisons. A *p* value of < 0.05 was considered statistically significant.

## 3. Results and Discussion

### 3.1. Programmed Death-Ligand 1 (PD-L1) Is Highly Expressed in Various Cancer Cells

**Figure 1:**
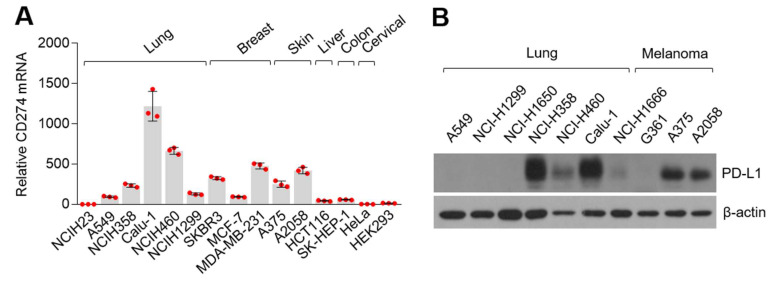
*CD274* and its translated protein PD-L1 expression in various cancer cells. (**A**) *CD274* mRNA expression in cultured cancer cell lines. *CD274* mRNA levels were measured by using quantitative real-time polymerase chain reaction (qRT-PCR) and expression levels were normalized to the human *36B4* gene. The values represent the mean ± SD. Experiments were performed in triplicates. Red dot indicates the value of the individual sample. (**B**) PD-L1 proteins in seven lung cancer and three melanoma cells were measured by using western blotting.

Although previous observational reports have demonstrated that statins improve therapeutic efficacy and the overall survival in patients with advanced NSCLC who were previously treated with nivolumab, programmed cell death protein 1 (PD-1)-neutralizing monoclonal antibodies [18,19], the precise molecular mechanism by which statins are associated with anti-cancer effects of immune checkpoint inhibitors targeting the interaction of PD-1 and programmed death-ligand 1 (PD-L1), such as nivolumab and pembrolizumab, have not been investigated. To investigate this, we initially measured PD-L1 protein and mRNA (*CD274*) levels in various cancer cells. Here, we found that PD-L1 encoding *CD274* mRNA levels were predominantly expressed in lung cancer (Calu-1 and NCI-H460), breast cancer (SKBR3 and MDA-MB-231), and melanoma (A375 and A2058) (Figure 1A). PD-L1 protein levels were highly expressed in NCI-H358, NCI-H460, and Calu-1 lung cancer, and in A375 and A2058 melanoma cells (Figure 1B). Similarly, previous reports have shown that PD-L1 protein levels are increased in various lung cancer and melanoma cells, including Calu-1, NCI-H460, A375, and A2058 [39,40,41]. Our observation indicates that lung cancer and melanoma cells are appropriate for investigating the functional role of statins in regulating PD-L1 expression in cancer cells.

### 3.2. Statins Suppress PD-L1 Expression in Lung Cancer and Melanoma Cells

**Figure 2:**
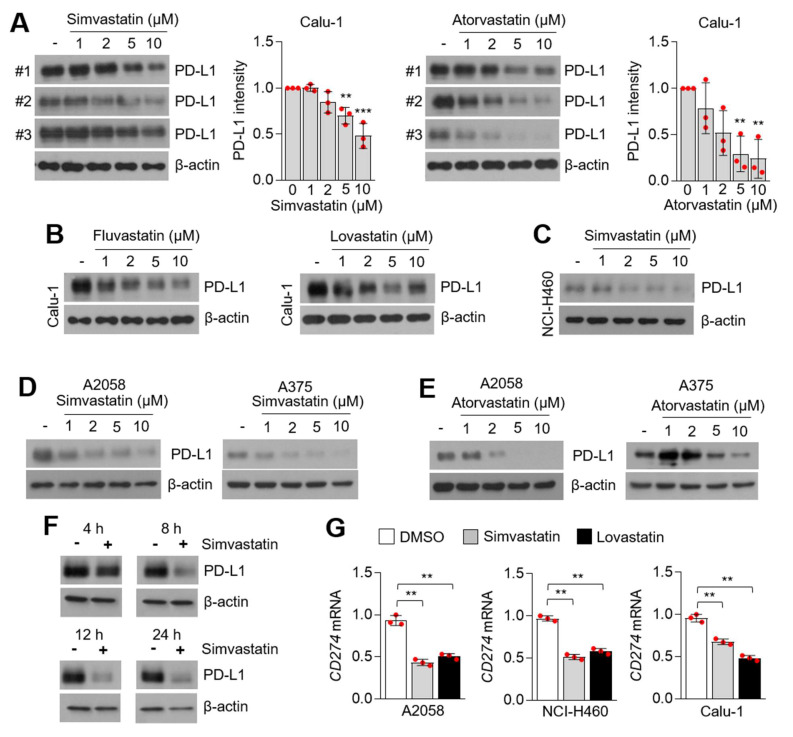
PD-L1 is decreased in statins-treated lung cancer and melanoma cells. (**A**) PD-L1 expression in simvastatin and atorvastatin-treated Calu-1 lung cancer cells. Calu-1 cells were incubated with simvastatin and atorvastatin at the indicated concentration for 12 h. DMSO was used for vehicle control (-). PD-L1 protein levels were measured by using western blotting and PD-L1 protein intensity was both quantified and represented by Image J and Graphpad Prism. Each PD-L1 protein level in the statins-treated sample was compared to the vehicle sample. The values represent the mean ± SD for the three independent experiments (#1, #2, and #3) performed. ** *p* < 0.01 and *** *p* < 0.001 by one-way ANOVA, followed by an appropriate post hoc test for the comparison between two experimental groups. (**B**) PD-L1 expression in fluvastatin and lovastatin-treated Calu-1 lung cancer cells. Calu-1 cells were incubated with fluvastatin and lovastatin at the indicated concentration for 12 h. (**C**) PD-L1 expression in simvastatin-treated NCI-H460 lung cancer cells. Different concentrations of simvastatin were treated for 12 h, as indicated. (**D**,**E**) PD-L1 expression in simvastatin (**D**) or atorvastatin (**E**)-treated A375 and A2058 melanoma cells. Different concentration of statins were treated for 12 h. (**F**) PD-L1 expression in simvastatin-treated A2058 melanoma cells in a time-dependent manner, as indicated. (**G**) CD274 mRNA-encoding PD-L1 protein expression in simvastatin (5 µM for 8 h) and lovastatin (5 µM for 8 h)-treated A2058, NCI-H460, and Calu-1 cells. CD274 mRNA was measured by using qRT-PCR. Values represent mean ± SD. Experiments were performed in triplicates. ** *p* < 0.01 by one-way ANOVA, followed by an appropriate post hoc test for the comparison between two experimental groups.

To evaluate the suppressive role of statins on PD-L1 expression, we applied treatment with four statins (simvastatin, atorvastatin, lovastatin, and fluvastatin) and measured PD-L1 protein expression in lung cancer and melanoma cells. Figure 2A shows that simvastatin and atorvastatin dose-dependently attenuated PD-L1 expression in Calu-1 cells (Figure 2A). In addition, fluvastatin and lovastatin also effectively decreased PD-L1 expression in Calu-1 cells (Figure 2B). In a similar manner, decreased PD-L1 protein levels were detected in simvastatin-treated NCI-H460 cells in a dose-dependent manner (Figure 2C). Simvastatin and atorvastatin also suppressed PD-L1 expression in A375 and A2058 melanoma cells in a dose-dependent manner (Figure 2D,E). To determine the time point when simvastatin was effective in reducing PD-L1 expression, simvastatin was administered for 4, 8, 12, and 24 h in A2058 cells. Here, we found that decreased PD-L1 protein levels were observed after 4 h of simvastatin treatment (Figure 2F). To understand whether statins suppress PD-L1 on the transcriptional or post-transcriptional levels, *CD274* mRNA-encoding PD-L1 protein expression was measured in melanoma (A2058) and lung cancer (NCI-H460 and Calu-1) cells. Decreased *CD274* mRNA levels were observed following statins treatment (Figure 2G). These results indicate that statins suppress PD-L1 expression on the transcriptional level in lung cancer and melanoma cells. Recent work has shown that niclosamide enhances T-cell cytotoxicity and anti-cancer effects of the PD-1/PD-L1 blockade by suppressing PD-L1 expression in non-small cell lung carcinoma (NSCLC) [42]. Similarly, regorafenib, a multi-kinase inhibitor, was found to enhance the anti-cancer effect of anti-PD-1 by activating CD8+ T-cells and suppressing IFNγ-induced PD-L1 expression in melanoma cells [43]. Considering that PD-L1-suppressive small molecules, such as niclosamide and regorafenib, have shown benefits against the efficacy of the PD-1/PD-L1 immune checkpoint blockade in NSCLC and melanoma [42,43], we hypothesized that statins could also be used for combination cancer therapy with immune checkpoint blockades. In addition, increased evidence has shown that statins suppress cancer cell growth in vitro and in vivo [44]. PD-L1 promotes cancer cell growth and silencing of PD-L1 by using short hairpin RNA (shRNA)-attenuated cancer cell growth in vitro and in vivo in both NSCLC and hepatocellular carcinoma (HCC) [45,46]. These previous observations also support our findings that suppression of PD-L1 by statins might be a possible molecular mechanism for the anti-cancer effects of statins.

### 3.3. Statins Suppress PD-L1 Expression Independent of Farnesyltransferases (FTase) and Geranylgeranyltransferases (GGTase)

**Figure 3:**
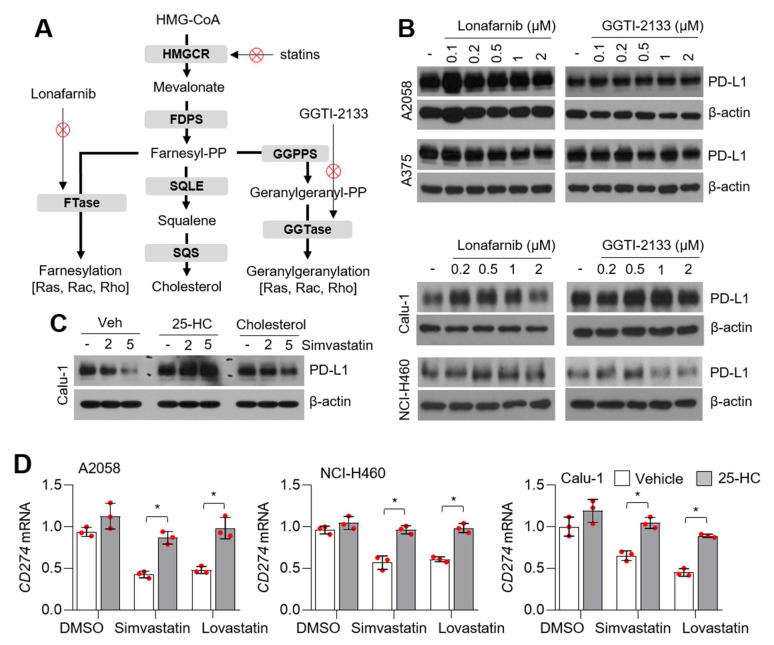
Intracellular cholesterol is associated with the suppression of PD-L1 in statins-treated lung cancer and melanoma cells. (**A**) Schematic representation of the mevalonate and cholesterol biosynthesis pathways. (**B**) Lonafarnib (a selective inhibitor of FTase) and GGTI-2133 (a selective inhibitor of GGTase) did not decrease PD-L1 expression. Lung cancer (Calu-1 and NCI-H460) and melanoma (A375 and A2058) cells were incubated with different concentrations of lonafarnib or GGTI-2133 for 12 h, as indicated. Protein levels were measured by using western blotting. (**C**) Supplement of cholesterol reversed the suppression of PD-L1 by statins. Calu-1 lung cancer cells were pre-incubated with 20 µM of 25-hydroxycholesterol (25-HC) and 0.5 mM of water soluble cholesterol for 6 h, and then cells were further incubated with 2 or 5 µM of simvastatin for 8 h. After drug treatment, protein levels were measured by using western blotting. (**D**) 25-HC reversed the suppression of CD274 by statins treatment. Cells were incubated for 6 h in the absence or presence of 25-HC (20 µM) and then cells were further incubated with 5 µM of simvastatin or lovastatin for 8 h. CD274 mRNA levels were measured by using qRT-PCR. Values represent mean ± SD. Experiments were performed in triplicates. * *p* < 0.05 by Student’s *t*-test for two experimental comparisons.

To determine how statins attenuate PD-L1 expression, the functional roles of farnesyltransferases (FTase) and geranylgeranyltransferases (GGTase) on PD-L1 expression were evaluated in lung cancer and melanoma cells (Figure 3A). In this study, we found that lonafarnib (FTase inhibitor) and GGTI-2133 (GGTase inhibitor) did not affect PD-L1 expression in a dose-dependent manner, indicating that FTase and GGTase do not suppress PD-L1 expression by statins in lung cancer and melanoma cells (Figure 3B). To evaluate whether intracellular cholesterol acts as a major cause of statin-mediated PD-L1 attenuation, PD-L1 expression was measured in the absence or presence of 25-hydroxycholesterol (25-HC) and water-soluble cholesterol in statin-treated Calu-1 lung cancer cells. Here, we found that simvastatin did not reduce PD-L1 expression in the presence of 25-HC or water-soluble cholesterol (Figure 3C). In addition, decreased *CD274* mRNA levels in simvastatin and lovastatin-treated lung cancer and melanoma cells were reversed by 25-hydroxycholesterol (25-HC) treatment (Figure 3D). These results revealed that intracellular cholesterol levels are important for maintaining PD-L1 expression. Lipid rafts, which contain high levels of cholesterol and sphingolipids in the cellular membrane, mainly regulate intracellular signal transduction cascades through membrane-bound signaling proteins [47]. Zhuang et al. showed that simvastatin attenuates tyrosine phosphorylation of lipid raft proteins and AKT phosphorylation, consequently causing cellular apoptosis [48]. Similarly, attenuated insulin receptor activation was observed to inhibit cholesterol biosynthesis in 3T3-L1 preadipocytes through the disruption of lipid rafts [49]. Given that cellular signal transduction cascades via lipid rafts are controlled by the intracellular cholesterol pool [48,49], we hypothesized that PD-L1 expression might be tightly regulated by cellular signal transduction cascades, such as the insulin receptor and AKT-signaling in cancer cells.

### 3.4. AKT-Signaling Involves PD-L1 Suppression by Statins

**Figure 4:**
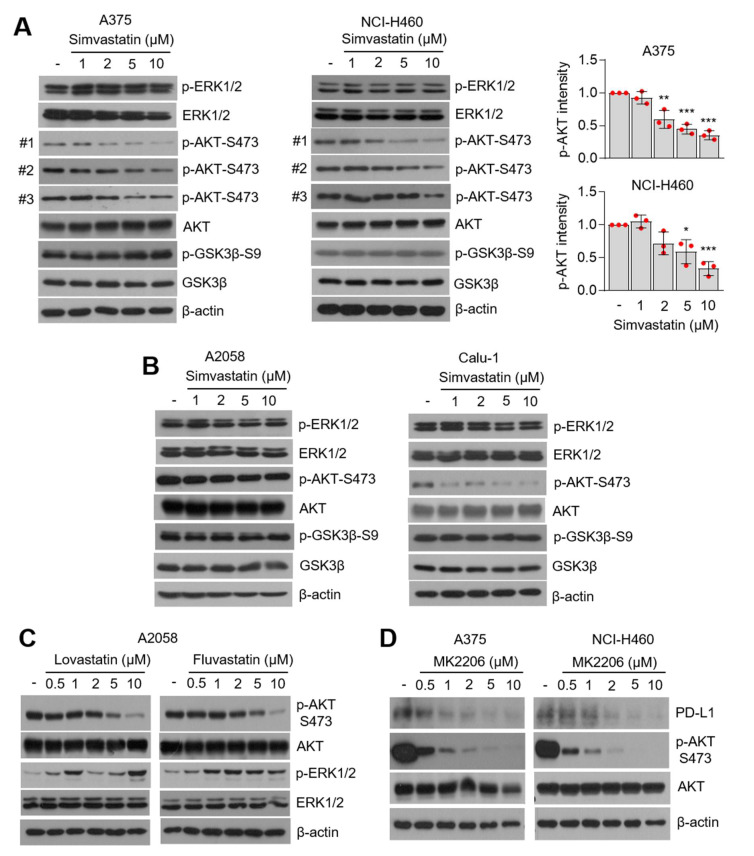
Inhibition of AKT-signaling involved PD-L1 suppression by statins. (**A**) Simvastatin decreased phosphorylation of AKT but not ERK1/2 and GSK3β. A375 and NCI-H460 cells were incubated with different concentrations of simvastatin for 12 h, as indicated. Phosphorylated AKT protein levels were measured by using western blotting. Phosphorylated AKT intensity was quantified and represented by Image J and Graphpad Prism. Each phosphorylated AKT protein level in the statins-treated sample was compared to the vehicle sample. The values represent the mean ± SD from the three independent experiments (#1, #2, and #3) performed. Values represent mean ± SD. * *p* < 0.05, ** *p* < 0.01 and *** *p* < 0.001 by one-way ANOVA, followed by an appropriate post hoc test for the comparison between two experimental groups. (**B**) Simvastatin suppressed AKT phosphorylation in A2058 and Calu-1 cells. (**C**) Lovastatin and Fluvastatin attenuated AKT phosphorylation. (**D**) A selective AKT inhibitor, MK2206, decreased PD-L1 expression in lung cancer (NCI-H460) and melanoma (A375) cells. DMSO as a vehicle or different concentrations of MK2206 were treated in NCI-H460 and A375 cells, and were incubated for 6 h. Protein levels were measured by using western blotting.

It is clear that oncogenic-signaling, including phosphoinositide 3-kinases (PI3K) and mitogen-activated protein kinase (MAPK), is attenuated by statins in multiple types of cancer cells [50,51,52,53,54]. Thus, phosphorylation of AKT and ERK1/2 was measured in statin-treated lung cancer and melanoma cells. Phosphorylation of AKT-S473 (phosphorylated form on 473 serine), but not GSK3β and ERK1/2, were downregulated in simvastatin-treated lung cancer (NCI-H460 and Calu-1) and melanoma (A375 and A2058) cells in a dose-dependent manner (Figure 4A,B). Additionally, lovastatin and fluvastatin attenuated AKT-S473 phosphorylation (Figure 4C). AKT and other kinases, such as p70RSK and PKC, phosphorylate GSK3β [55]. Here, we also found that GSK3β was not affected by statins, even in those with a suppression of AKT phosphorylation (Figure 4A,B). Consistently, PI3K-AKT-independent but mTORC1-dependent phosphorylation of GSK3β has been reported [56]. Figure 4D shows that a selective inhibitor of AKT (MK2206) dose-dependently reduced PD-L1 expression in lung cancer (NCI-H460) and melanoma (A375) cells. Consistent with our results, a previous report showed that MK2206 and rapamycin (an inhibitor of mTOR) suppress PD-L1 expression in glioblastoma and triple-negative breast cancer (TNBC) cells [38,57]. Thus, we demonstrated that PD-L1 expression is regulated by the PI3K-AKT-signaling pathway in lung cancer, melanoma, glioblastoma, and breast cancer. In the present study, our results revealed that statins attenuate phosphorylation of AKT, but not GSK3β and ERK1/2, in lung cancer and melanoma cells. In contrast, simvastatin and lovastatin were found to inhibit the Raf-MEK-ERK1/2-signaling cascade by decreasing farnesyl pyrophosphate (FPP) and geranylgeranyl pyrophosphate (GGPP)-mediated protein prenylation in leukemia and TNBC cells [54,58]. Thus, we speculated that statin-induced attenuation of oncogenic growth-signaling is dependent on the cellular type and lipid metabolic flexibility.

### 3.5. Statins Suppress β-Catenin Phosphorylation

**Figure 5:**
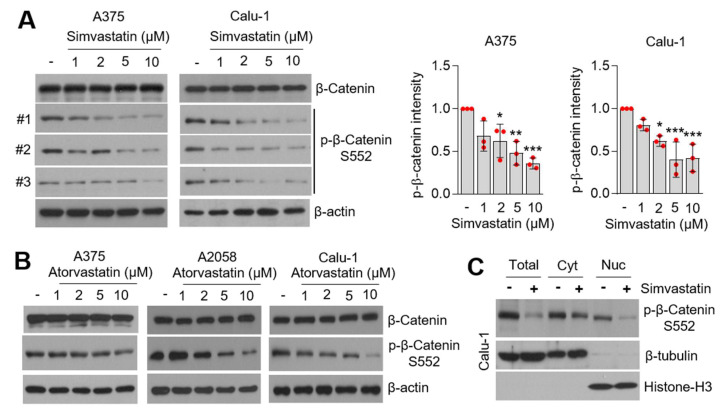
Statins decreased phosphorylation of β-catenin-S552. (**A**) Suppression of β-catenin-S552 phosphorylation in simvastatin-treated lung cancer (Calu-1) and melanoma (A375) cells. Cells were incubated with different concentrations of simvastatin for 6 h. Protein expression was confirmed by using western blotting. Phosphorylated β-catenin protein levels were measured by using western blotting. Phosphorylated β-catenin intensity was quantified and represented by Image J and Graphpad Prism. Each phosphorylated β-catenin-S552 protein level in the statins-treated sample was compared to the vehicle sample. The values represent the mean ± SD from the three independent experiments (#1, #2, and #3) performed. Values represent mean ± SD. * *p* < 0.05, ** *p* < 0.01 and *** *p* < 0.001 by one-way ANOVA, followed by an appropriate post hoc test for the comparison between two experimental groups. (**B**) Atorvastatin suppressed β-catenin-S552 phosphorylation in A375, A2058, and Calu-1 cells. (**C**) Simvastatin decreased nuclear β-catenin. Cells were incubated with DMSO (as a vehicle control) or simvastatin (10 µM) for 8 h and nuclear β-catenin protein levels were measured by using western blotting.

Multiple types of transcription factors, such as hypoxia-inducible factor 1 alpha (HIF1α), nuclear factor-κB (NF-κB), MYC, the signal transducer and activator of transcription 3 (STAT3), and β-catenin, are known to increase *CD274* expression in response to hypoxia, inflammatory cytokines, the epidermal growth factor (EGF), and Wnt-signaling [23]. Du et al. showed that activation of AKT-S473, in response to the Wnt ligand and activated epidermal growth factor receptor (EGFR), phosphorylates and activates β-catenin-S552 (phosphorylated form of 552 serine residue), and consequently the β-catenin-S552/TCF4/LEF1 complex promotes both *CD274* expression and immune evasion in glioblastoma [38]. Considering we found that statins suppress phosphorylation of AKT-S473 (Figure 4), we speculated that β-catenin-S552 was associated with statin-mediated PD-L1 attenuation. Significant decreased phosphorylation of β-catenin-S552 was observed in simvastatin-treated lung cancer (Calu-1) and melanoma (A375) cells in a dose-dependent manner (Figure 5A). Consistent with this result, 10 µM of atorvastatin was also found to suppress β-catenin-S552 in lung cancer and melanoma cells (Figure 5B). Figure 5C shows that simvastatin decreased phosphorylated β-catenin-S552 in the nuclear extract, indicating that statins might decrease the transcriptional activity of β-catenin. In addition, the total form of β-catenin was not altered in statins-treated cells (Figure 5A,B). These results are in line with that statins do not decrease phosphorylation of GSK3β, suggesting that the suppression of β-catenin-S552 in statins-treated lung cancer and melanoma cells may be independent of both GSK3β-mediated phosphorylation and proteasomal degradation of β-catenin.

### 3.6. Statins Suppress PD-L1 at the Transcriptional Level

**Figure 6:**
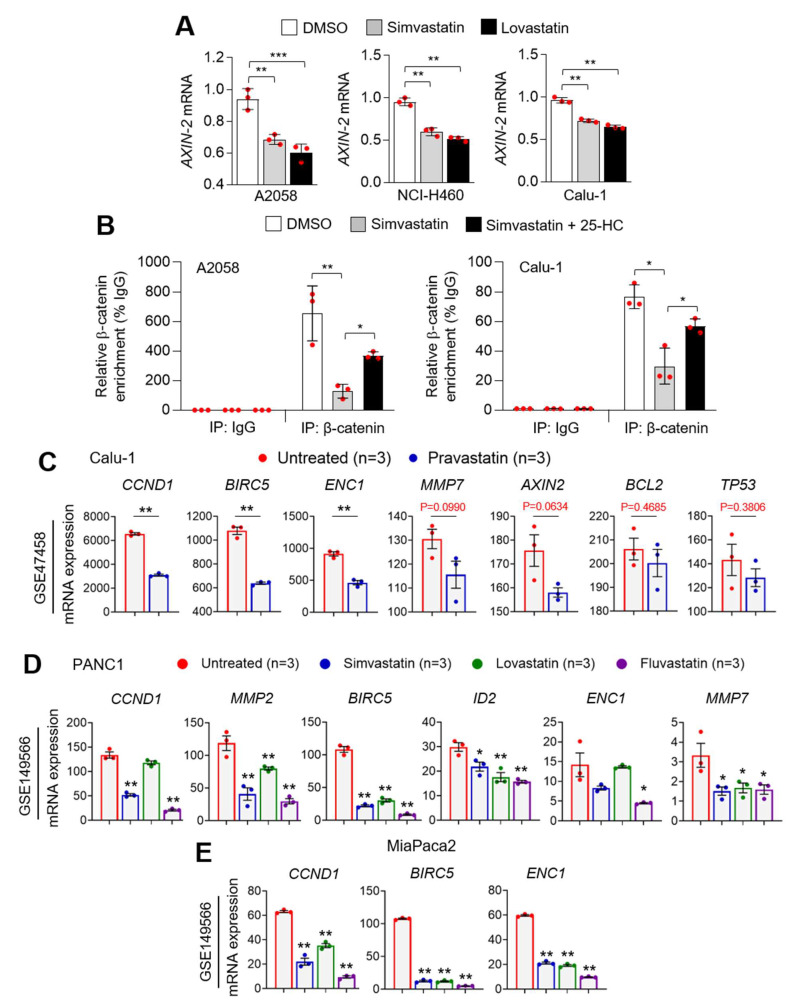
Statins decreased β-catenin target genes. (**A**) Simvastatin and lovastatin attenuated AXIN-2 mRNA, as a β-catenin target gene, in lung cancer and melanoma cells. Lung cancer (NCI-H460 and Calu-1) and melanoma (A2058) cells were incubated with simvastatin (5 µM) or lovastatin (5 µM) for 6 h, as indicated. AXIN-2 was measured by using qRT-PCR. Experiments were performed in triplicates. All plots indicate the mean ± SD. ** *p* < 0.01 and *** *p* < 0.001 by one-way ANOVA, followed by an appropriate post hoc test for the comparison between two experimental groups. (**B**) Chromatin immunoprecipitation (ChIP) for β-catenin followed by ChIP-qPCR in A2058 and Calu-1 cells confirmed a chromatin enrichment of β-catenin at the CD274 promoter loci (600 bp upstream from the transcription start site). Cells were incubated for 6 h in the absence or presence of 25-HC (20 µM) and then cells were further incubated with 5 µM of simvastatin for 8 h. All plots indicate the mean ± SD. Experiments were performed in triplicates. * *p* < 0.05 and ** *p* < 0.01 by one-way ANOVA, followed by an appropriate post hoc test for the comparison between two experimental groups. (**C**) β-catenin target genes, such as CCND1, BIRC5, ENC1, MMP7, AXIN-2, BCL2, and TP53, mRNA expression levels were measured in pravastatin-treated Calu-1 cells using a publicly available microarray dataset (GSE47458). All plots indicate the mean ± SD. ** *p* < 0.01 by Student’s *t*-test for two experimental comparisons. (**D**,**E**) CCND1, MMP2, BIRC5, ID2, ENC1, and MMP7 mRNA levels were measured in simvastatin, lovastatin, or fluvastatin-treated (**D**) PANC1 and (**E**) MiaPaca2 pancreatic cancer cells using a publicly available microarray dataset (GSE149566). All plots indicate the mean ± SE. * *p* < 0.05 and ** *p* < 0.01 by one-way ANOVA, followed by an appropriate post hoc test for the comparison between two experimental groups.

Considering phosphorylated and activated β-catenin-S552 promotes *CD274* expression at the transcriptional level [23], we further measured *AXIN-2* expression, which is a well-known β-catenin target gene, in statin-treated lung cancer and melanoma cells. Consistent with the suppression of *CD274* by statins, decreased *AXIN-2* mRNA was observed following statins treatment (Figure 6A). In addition, we found that simvastatin decreases the *CD274* promoter occupancy of β-catenin, which was reversed by supplementation of 25-HC in lung cancer (Calu-1) and melanoma (A2058) cells (Figure 6B). To strengthen our observation, various β-catenin target genes were analyzed in statins (pravastatin, simvastatin, lovastatin, and fluvastatin)-treated lung (Calu-1) and pancreatic (PANC1 and MiaPaca2) cancer cells using publicly available datasets. Pravastatin was found to significantly decrease β-catenin target genes, such as *CCND1*, *BIRC5*, and *ENC1*, in Calu-1 lung cancer cells (Figure 6C). Similarly, decreased various β-catenin target genes were also observed in simvastatin, lovastatin, or fluvastatin-treated PANC1 and MiaPaca2 pancreatic cancer cells (Figure 6D,E). These findings indicate that phosphorylated and activated β-catenin-S552 via AKT-S473 involves statin-mediated PD-L1 suppression in lung cancer and melanoma cells.

## 4. Conclusions

When viewed as a comprehensive study, our observations could provide an understanding of the molecular mechanisms by which statins have beneficial effects for improving the therapeutic efficacy of immune checkpoint inhibitors targeting PD-1 and its ligand PD-L1. Furthermore, extended preclinical and clinical evaluations based on these findings could help determine whether statins could improve the anti-cancer efficacy of immune checkpoint inhibitors and may be useful to expand the clinical practice guidelines for the treatment of lung cancer and melanoma therapy.

## Figures and Tables

**Table 1:** Primer sequences for quantitative real time-PCR.

Gene	Forward Primer	Reverse Primer
Axin-2	GAGTGGACTTGTGCCGACTTCA	GGTGGCTGGTGCAAAGACATAG
CD274	CTGCACTTTTAGGAGATTAGATC	CTACACCAAGGCATAATAAGATG
36B4	CATGTTGCTGGCCAATAAGG	TGGTGATACCTAAAGCCTGGAA
